# From Neck Pain to Sarcoidosis: The Interesting Association

**DOI:** 10.1155/2021/6663519

**Published:** 2021-03-16

**Authors:** Fadi Kharouf, Mohammad Yassin, Issa Al-kharouf, Issam Hindi, Rottem Kuint

**Affiliations:** ^1^Department of Medicine, Rheumatology Unit, Hebrew University-Hadassah Medical Center, Jerusalem, Israel; ^2^Diagnostic Radiology Unit, Maccabi Healthcare Services, Jerusalem, Israel; ^3^Department of Pathology, Hebrew University-Hadassah Medical Center, Jerusalem, Israel; ^4^Department of Medicine, Institute of Pulmonary Medicine, Hebrew University-Hadassah Medical Center, Jerusalem, Israel

## Abstract

We report the case of a 31-year-old male patient, presenting to the emergency department (ED) with a 6-week history of left-sided lateral neck pain, along with a minor localized swelling. A few weeks after the beginning of his complaints, he contracted a mild coronavirus disease 2019 (COVID-19). Upon examination, his aches were defined as carotidynia; thus, proper radiologic evaluation was carried out. While ultrasound (US) and magnetic resonance imaging (MRI) scans showed evident signs of left common carotid (LCC) vasculitis, computed tomography angiography (CTA) and positron emission tomography-CT (PET-CT) scans revealed no vascular findings. Unexpected hypermetabolic hilar and mediastinal lymphadenopathy was found on PET-CT, necessitating lymph node biopsy. Pathology results displayed noncaseating granulomas. Besides, angiotensin-converting enzyme (ACE) levels in blood were high. Sarcoidosis, with concurrent LCC vasculitis, was diagnosed, and corticosteroid therapy was started. Shortly thereafter, remarkable recovery ensued.

## 1. Introduction

Sarcoidosis is an inflammatory disease that may impact any organ system. Noncaseating granulomas have long been known as its pathological hallmark. The clinical spectrum of the disease is diverse, ranging from asymptomatic radiographic findings, to progressive multiorgan failure [1]. Vasculitis has been rarely described as a feature of, or in association with, sarcoidosis [2, 3]. When present, it most commonly correlates with pulmonary sarcoidosis and may affect any vessel size [2]. It can mimic other vasculitides, including microscopic polyangiitis, polyarteritis nodosa, and Takayasu's disease (TAK). Corticosteroids and cytotoxic drugs are the mainstay of therapy, but relapses are common [3].

Here, we present a unique case of coexisting sarcoidosis and vasculitis. We discuss the clinical manifestations, diagnostic challenges, and therapeutic aspects.

## 2. Case Presentation

A 31-year-old male patient presented to the emergency department (ED) with a 6-week history of left-sided lateral neck pain, accompanied by a minor localized swelling. He denied throat aches, dyspnea, fever, weight loss, or other constitutional symptoms.

Three weeks after the appearance of the neck pain, the patient contracted a mild coronavirus disease 2019 (COVID-19), manifesting with anosmia and ageusia. The latter symptoms improved within several days, but the neck pain persisted throughout the whole period. The patient's past medical history was otherwise unremarkable.

**Figure 1:**
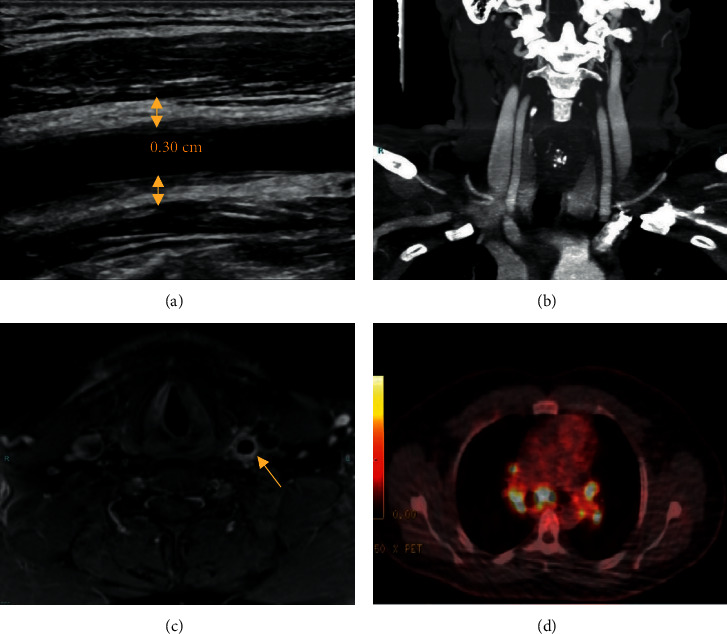
(a) Longitudinal ultrasound view showing LCC wall thickening (arrows). 0.30 cm relates to vessel wall thickness. (b) Coronal CTA view showing no LCC wall thickening. (c) Axial MRI view showing prominent LCC wall thickening. (d) PET/CT scan showing hypermetabolic mediastinal and hilar lymphadenopathy. ^∗^Dimensions not to scale. CTA : computed tomography angiography; LCC : left common carotid; PET : positron emission tomography; MRI : magnetic resonance imaging.

On physical examination, prominent tenderness was noted over the left common carotid (LCC) artery, with subtle swelling. Upper extremity pulses were symmetrical. While blood counts, serum creatinine, and erythrocyte sedimentation rate were within normal limits (WNLs), C-reactive protein (CRP) was mildly elevated (Table 1). Doppler ultrasound (US) displayed evident LCC wall thickening, without luminal stenosis (Figure 1(a)). Head and neck computed tomography angiography (CTA) scan, however, showed no vasculitic lesions (Figure 1(b)). In light of the laboratory and CTA results, the patient was discharged, with the diagnosis of vasculitis being erroneously deferred.

**Figure 2:**
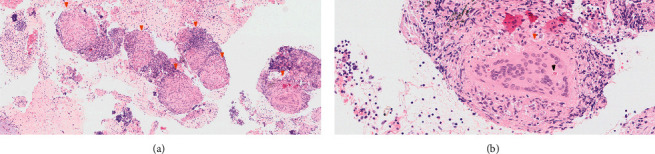
(a) Numerous noncaseating sarcoid-like granulomas (orange arrowheads). (b) Well-formed granuloma (orange arrowheads) with a developing Schaumann body (black arrowhead).

A repeat US performed two days later, per the radiologist's insistence, confirmed significant LCC wall thickening, thus, the patient was again referred to the ED for further evaluation. This time, he was hospitalized, and a head and neck magnetic resonance imaging (MRI) scan was completed, showing prominent LCC wall thickening, compatible with vasculitis (Figure 1(c)). Antinuclear antibodies (ANAs), anti-neutrophil cytoplasmic antibodies (ANCAs), and immunoglobulin levels, including immunoglobulin G4 (IgG4), were all unremarkable (Table 1). Serologies for syphilis and human immunodeficiency virus (HIV) were negative. In light of these findings, positron emission tomography (PET)-CT was carried out. While no active vasculitis was observed, hypermetabolic mediastinal and hilar lymphadenopathy was seen (Figure 1(d)). As such, bronchoscopy with endobronchial US transbronchial needle aspiration (EBUS-TBNA) was performed, and mediastinal lymph nodes (LNs) were biopsied. Pathology results demonstrated noncaseating granulomas (Figure 2), with negative acid fast and silver stains. Immunohistochemical staining for IgG4 was also negative. Further evaluations, including electrocardiography, transthoracic echocardiography, ophthalmologic check-up, pulmonary function tests, urinalysis, and urinary calcium levels, were all unremarkable. Angiotensin-converting enzyme (ACE) levels in blood were elevated, however. The typical radiologic and pathological features led to the diagnosis of sarcoidosis.

**Figure 3:**
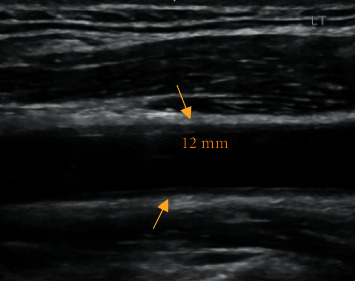
Longitudinal ultrasound view showing a noticeable improvement in LCC wall thickening (arrows) after corticosteroid therapy, as compared to Figure 1(a). 0.12 cm relates to vessel wall thickness. ^∗^Dimensions not to scale. LCC : left common carotid.

Prednisone, 40 mg, was started, leading to immediate resolution of carotidynia. Repeat neck US, within 6 weeks of therapy, revealed improvement of the sonographic findings (Figure 3). Likewise, a chest CT scan, performed within 4 months, showed diminution of the known hilar and mediastinal lymphadenopathy.

## 3. Discussion

Our patient was diagnosed with sarcoidosis and large vessel vasculitis (LVV). While several case reports and series have described the concurrence of the two conditions, the etiopathogenetic relation between them remains undefined. Similarities between these cases include the observation that sarcoidosis is often diagnosed first, with a usual lag period of several years. Another noteworthy finding is the frequent occurrence of uveitis. Furthermore, the aorta and its major branches are commonly involved, and the response to corticosteroid therapy appears favorable [4].

While the main clinical suspicion in our patient was of TAK, normal arterial findings on the CTA scan have cast doubt on such a diagnosis. CTA enables the visualization of the vessel wall and luminal changes [5]. Only a single small study was reported for CTA in TAK, yielding a sensitivity and specificity of 100%, using conventional angiography as the reference [6]. According to the most recent European League against Rheumatism (EULAR) recommendations, MRI is considered the first imaging test to diagnose TAK, through investigating mural inflammation and luminal changes. While PET may be valuable in detecting alternative diagnoses in patients with unspecific symptoms, US may be useful in cases of limb claudication, where carotid vasculitis may be elucidated [5].

Severe acute respiratory syndrome coronavirus 2 (SARS-CoV-2) may cause endothelial cell inflammation, thus culminating in vascular events [7]. In fact, Oda et al reported a case of adult LVV associated with COVID-19 [8]. While mediastinal lymphadenopathy is not a typical radiologic feature of COVID-19, it has been reported in association with the disease [9]. As aforementioned, our patient experienced only a mild viral illness. The fact that carotidynia appeared a few weeks prior to his SARS-CoV-2 infection and the lack of radiologic parenchymal findings render the association with COVID-19 unlikely.

In summary, we believe that the rarely reported concurrence of sarcoidosis and LVV, the striking discrepancy between the vascular findings in imaging tests, and the favorable response to corticosteroid therapy are all remarkable aspects of our case.

## Figures and Tables

**Table 1:** The patient's blood tests during admission.

Lab parameter	Value
Creatinine, micromol/L (62–115)	85
Calcium, mmol/L (2.08–2.65)	2.23
Albumin, g/L (32–48)	40
Aspartate transaminase, U/L (0–34)	**39** ^∗^
Alkaline phosphatase, U/L (46–116)	84
Lactate dehydrogenase, U/L (120–246)	210
Leukocytes/*μ*L (3,790–10,330)	7,100
Hemoglobin, g/dL (13.9–17.7)	15.5
Platelets/*μ*L (166,000–389,000)	225,000
ESR, mm/hr (1–20)	13
CRP, mg/dL (0–0.5)	**1**
Immunoglobulin G, mg/dL (700–1700)	1560
IgG4, mg/dL (4–86)	62.7
ACE, *μ*g/L (10–52)	**103**
ANA	Negative
c-ANCA	Negative
p-ANCA	Negative

Normal range values appear in brackets. Abnormal results appear in bold. ^∗^Mildly elevated for years. ESR : erythrocyte sedimentation rate; CRP : C-reactive protein; IgG4 : immunoglobulin G4; ACE : angiotensin-converting enzyme; ANA : antinuclear antibody; c-ANCA : cytoplasmic anti-neutrophilic cytoplasmic antibody; p-ANCA : perinuclear ANCA.

## Data Availability

Data can be made available through contact with the corresponding author, Fadi Kharouf, through fadikharouf@hotmail.com.

## Abbreviations

ACE: Angiotensin-converting enzyme
ANA: Antinuclear antibody
ANCA: Anti-neutrophil cytoplasmic antibody
COVID-19: Coronavirus disease 2019
CRP: C-reactive protein
CT: Computed tomography
CTA: Computed tomography angiography
EBUS-TBNA: Endobronchial ultrasound transbronchial needle aspiration
ED: Emergency department
EULAR: European league against rheumatism
HIV: Human immunodeficiency virus
IgG4: Immunoglobulin G4
LCC: Left common carotid
LN: Lymph node
LVV: Large vessel vasculitis
PET: Positron emission tomography
MRI: Magnetic resonance imaging
SARS-CoV-2: Severe acute respiratory syndrome coronavirus 2
TAK: Takayasu's disease
US: Ultrasound
WNLs: Within normal limits.
